# Cave dogs around major urban areas of Arequipa, Peru, threaten rabies elimination program

**DOI:** 10.3389/fvets.2025.1649737

**Published:** 2025-11-19

**Authors:** Ricardo Castillo-Neyra, Elvis W. Díaz, Brinkley Raynor Bellotti, Katherine Morucci, Micaela De la Puente-León, Lizzie Ortiz-Cam, Michael Z. Levy

**Affiliations:** 1 Department of Biostatistics, Epidemiology, and Informatics, University of Pennsylvania, Philadelphia, PA, United States; 2 School of Veterinary Medicine, University of Pennsylvania, Philadelphia, PA, United States; 3 One Health Unit, School of Public Health and Administration, Universidad Peruana Cayetano Heredia, Lima, Peru; 4 Department of Internal Medicine - Infectious Diseases, Wake Forest University School of Medicine, Winston-Salem, NC, United States; 5 Dry Forest Conservation Programme (Drycop) - ONG ConservAcción, Lima, Peru; 6 School of Health Sciences, Universidad San Ignacio de Loyola, Lima, Peru

**Keywords:** dog population dynamics, feral dogs, one health, periurban ecology, rabies, zoonoses

## Abstract

**Background:**

In the city of Arequipa, Peru, the government has implemented control measures against dog rabies virus since the detection of its reintroduction in 2015. The city was previously considered free of animal reservoirs, except for free-roaming owned dogs, animals with identifiable owners but allowed to move unsupervised, and stray dogs, which include both abandoned and street-born dogs that roam freely while relying on human settlements for food, within its urban boundaries. However, multiple reports from peri-urban residents have suggested the presence of feral dogs, a population living independently of humans on the city's outskirts. We aim to document the presence and dietary patterns of feral dogs adjacent to the city margins.

**Methods:**

We conducted monthly field visits to four peri-urban localities in eastern Arequipa, an area where the presence of feral dogs had been previously reported. Dog caves were identified by tracking footprints and other field signs left by dogs, and their locations were georeferenced. Each cave was revisited monthly three times to record the presence of live and dead dogs, and puppies. Fecal samples collected around the caves were analyzed to assess dietary patterns.

**Results:**

We observed that feral dogs use caves for resting, hiding, and reproduction—some of which appear to be constructed by the dogs themselves. The high number of puppies and dead adult dogs indicates a high population turnover. Dietary analysis revealed that these dogs feed on local fauna, including birds, rodents, cats, sheep, and, notably, other dogs.

**Conclusions:**

These unowned, cave-dwelling dogs are not reached by mass rabies vaccination or sterilization programs. Moreover, they exist outside the jurisdiction of health inspectors responsible for rabies surveillance, resulting in a lack of data on rabies infection in this subpopulation. Our findings highlight the need for integrated One Health strategies to address the potential challenges posed by feral dog populations in rabies elimination efforts.

## Introduction

The canine rabies elimination program in the city of Arequipa, Peru faces a unique threat that has hitherto been unreported. The geographic positioning of Arequipa amidst the climatically unforgiving Andean high desert was believed to shield the urban fauna (1) from contact with local wildlife disease reservoirs (2). Since the reintroduction of the rabies virus in Arequipa, 394 rabid dogs have been detected, averaging nearly one case per week in most years. As a result, present rabies control efforts, like those in most Latin American countries (3, 4), have focused mostly on populations of owned dogs (those with human caretakers, whether confined or allowed to roam) (1). Control efforts have included a combination of annual, city-wide mass vaccination campaigns, localized vaccination rings around positive cases, passive surveillance of dog populations, as well as methods that have been proven around the world to be ineffective, such as sporadic culling of free-roaming dogs (5, 6). Despite these efforts, cases have continued to spread over the past seven years (7). The scope of current control paradigms would be insufficient to address the complexity of the local disease ecology if feral dogs (1) are present in Arequipa.

Feral dog populations have broad negative impacts, from diminishing environmental quality (8–10), to displacing native wildlife (11, 12), to affecting the locals' livelihoods by targeting livestock (8, 13). Most importantly, they pose a significant health risk as potential vectors to a variety of pathogens that affect wildlife, domestic animal species, and human populations (14), such as canine rabies and distemper viruses. Aside from the human impact of rabies and the potential role of feral dogs in the virus transmission, there is also the risk of feral dogs introducing the rabies virus to vulnerable wildlife populations (10, 14, 15), such as the common Andean fox. The establishment of a new native vector and the potential for a novel rabies virus transmission cycle—such as the historical spillover from domestic dogs to red foxes in Europe (16)—demonstrates how such shifts can complicate efforts to eliminate the disease from a given region.

Recent accounts from peri-urban dwelling inhabitants have detailed numerous sightings and occurrences in which “cave dogs from the hills” have come into the periphery of the city to hunt local domestic animals, including adult pigs and sheep. Herders of medium-sized livestock have reported packs of dogs jumping fences, destroying corrals, and killing farm animals. The presence of feral, cave-dwelling dogs in peri-urban areas on the city's margins may represent an overlooked challenge for rabies control. Because unowned dogs are excluded from vaccination and sterilization campaigns, and dogs outside city boundaries are not systematically monitored by rabies surveillance systems (3), important knowledge gaps remain regarding their potential role in rabies transmission dynamics. Surveillance and understanding of the behavioral ecology and extent of interactions between populations of owned and unowned dogs in the region are essential to explain the potential contribution of unowned dogs to the local propagation of the canine rabies virus (17–19). In this context, characterizing the feral cave-dogs' diet provides insight into their ecological role, their interactions with humans, domestic animals, and wildlife, and the resources that sustain them. Diet directly reflects whether these dogs rely primarily on anthropogenic sources (garbage, backyard livestock) or on hunting/scavenging of domestic and wild animals (20). This distinction has important epidemiological implications: scavenging and predation increase the frequency of direct contact with unvaccinated dogs, cats, and livestock, thereby expanding potential rabies transmission networks across populations that are otherwise considered distinct (21).

In response to these growing concerns, our objectives were to establish the presence of feral dogs in peri-urban areas of Arequipa and examine the potential challenges these animals pose for rabies control programs, as well as the broader One Health implications for local wildlife conservation and the sustainability of small-scale livestock farming.

## Materials and methods

### Definitions

Throughout this study, we used the following working definitions based on Beaver's descriptions of dog populations (1). *Owned dogs* are animals under human care, whether fully confined or allowed to roam freely. *Stray dogs* include formerly owned animals that have been abandoned as well as dogs born on the streets; they roam freely without an owner but typically remain partially dependent on human-derived food sources. *Feral dogs* live independently of humans, surviving in natural or remote environments, and generally avoid human contact.

### Study site

The study was conducted in the Alto Selva Alegre (ASA) district, located in the northeastern part of Arequipa, Peru, at an altitude of 2,200 masl. Arequipa city is the second largest city of Peru with a human population of ~1.1 million. ASA is a medium-sized (~7 km^2^) and diverse district that includes both densely populated urban areas and less developed peri-urban and natural zones. Its southeastern region borders the foothills of the Misti volcano, a rugged and sparsely inhabited area where sightings and reports of feral dogs have previously occurred. The district shares boundaries with the districts of Cayma, Miraflores, Yanahuara, and Arequipa Cercado.

### Field surveys

Between September and December 2019, we conducted monthly pedestrian surveys, systematically walking along trails within the study area (≈1.6 km^2^) to record direct field observations of free-roaming dogs in the northeastern portion of ASA. Our study area comprised three contiguous subdistrict localities—Apsil, El Roble, and San Luis A. The primary goal of these surveys was to identify caves that showed evidence of use by feral dogs and obtain parameters to characterize dogs and caves. A cave was defined as a naturally occurring or canine-modified dwelling in the ground or an earthen wall that was at least 1 meter in-depth, or large enough to permit animal entry and shelter, and also exhibited direct or indirect evidence of dog usage. Caves that were less than a meter in depth, but contained some evidence of dog usage, were georeferenced and recorded as a potential sleeping sight, rather than occupation. In 2022, we revisited the same three localities to collect fecal samples from in and around the identified caves. The goal of this second phase was to perform a dietary analysis to better understand the feeding behavior and ecological role of feral dogs in the area.

### Trails and mapping

Andean foxes and pumas live in Arequipa, so it was important to prevent misclassification of pawprints with these two species. Pawprints from the three species can be distinguished based on size, shape, and claw marks. Dog footprints from medium and large dogs, such as those observed in our study area, typically leave prints ranging from 7 to 10 cm in length. Dog prints show visible claw marks and a relatively broad, rounded pad. Andean fox footprints are noticeably smaller, usually 4–5 cm in length, with narrower toes, less prominent claw marks, and a more elongated pad compared to dogs. Puma footprints are larger, often exceeding 10 cm, and are characterized by a round shape with no visible claw marks and a distinct three-lobed rear pad impression. These differences allowed our personnel to reliably distinguish between the three species in the field.

During the initial reconnaissance visit, we observed a network of trails formed by the repeated movement of dogs (Figure 1A). This observation informed our decision to explore the use of these trails as a strategy to locate caves. To determine the most effective survey approach, we conducted a short pilot study comparing two methods. In the first, two surveyors followed a predefined grid-based transect, with lines spaced approximately 70 meters apart to form a regular grid. In the second method, two surveyors followed dog-made trails—paths visibly worn by repeated canine use—allowing the natural layout of the trails to guide the search. In one locality, the grid-based transect approach led to the identification of six dog-occupied caves. In contrast, the dog-trail-based approach identified all six of those same caves, along with 17 additional ones, for a total of 23. Based on these results, we selected the dog-trail-based method for the full survey.

**Figure 1A:**
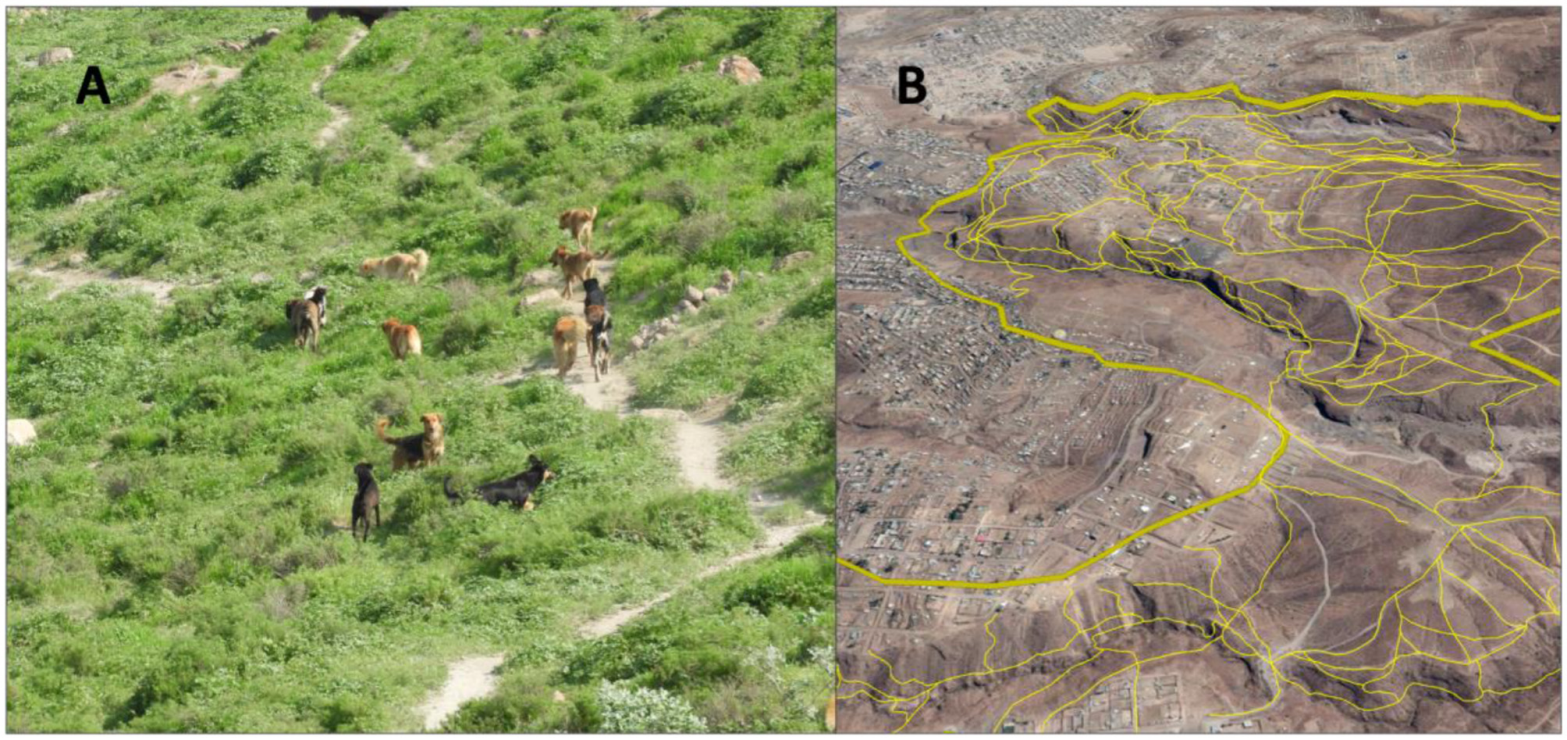
**(A)** Pack of dogs in study area leaving a clear path on frequently used tracks. Visible paths were used to determine the transects to search for cave dogs. **(B)** The thick yellow line delimits the study area, and thinner yellow lines represent transects followed by our team.

Using Google Earth, we mapped the visible footpaths created by dogs across the study area (Figure 1B) and used these as the basis for our transects. During subsequent visits, paired observers conducted foot surveys along these mapped trails, recording sightings of feral dogs and caves showing evidence of occupation or use.

**Figure 1B:**
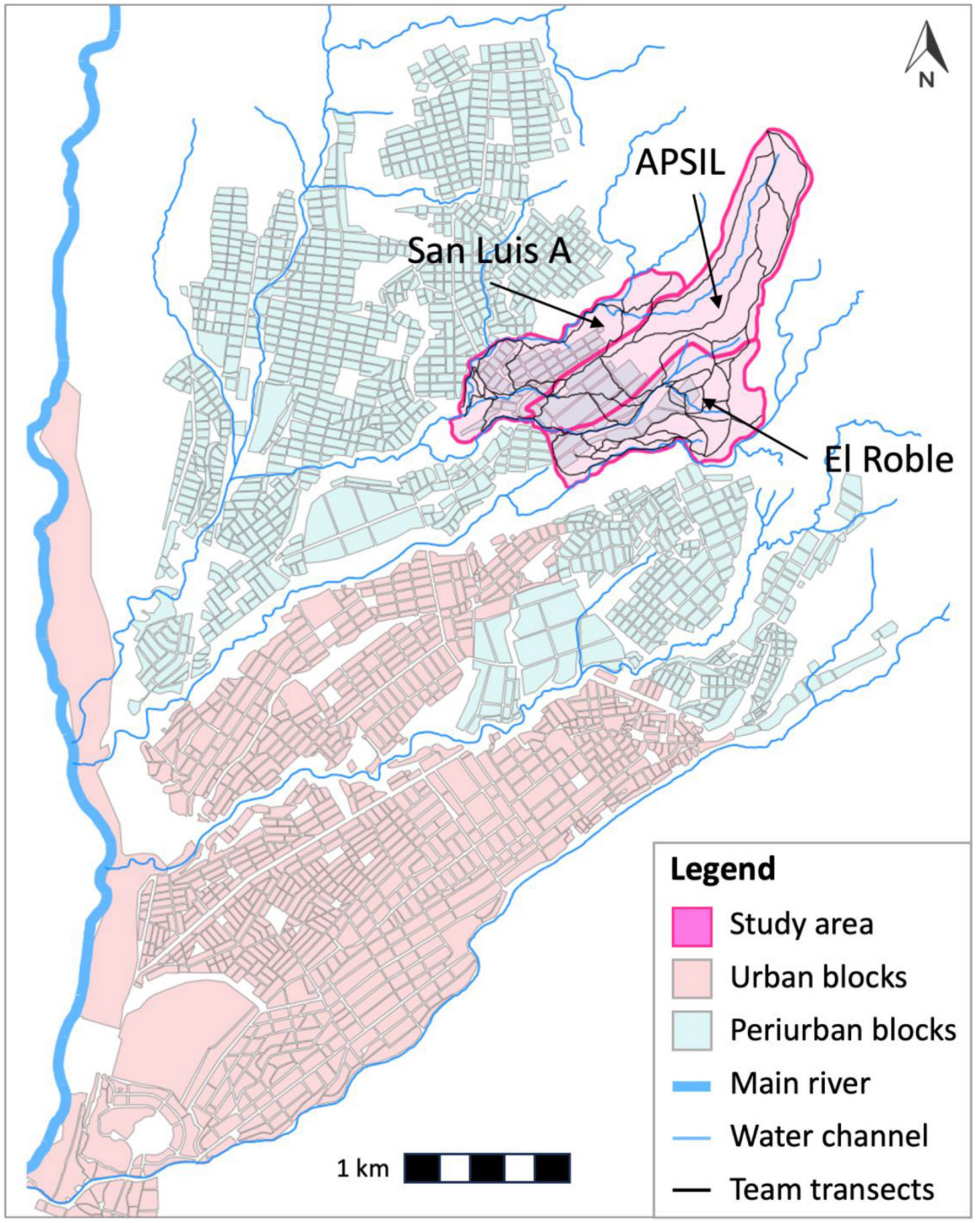
Map of the study area in the periurban region of Arequipa, Peru. The study area includes the localities of San Luis A, APSIL, and El Roble.

The region is connected to the rest of the city by a series of semi-natural water channels that serve to collect and deposit rainwater runoff into the city river during the wet season (2, 22). Given the short duration of the wet season, water channels are empty and dry during much of the year and are used by dogs to facilitate movement across city localities and throughout the study region (2, 22). We also visited the water channels and their surroundings in our study area.

### Monthly visitations and data collection

During the first site visitation, we recorded and georeferenced observations of living and dead dogs along with evidence of dog-associated caves. Foot surveys were conducted by a pair of observers along study trails on a monthly basis thereafter.

*Direct evidence* was limited to the sighting of adult or juvenile dogs either in the cave itself or in the direct vicinity. *Indirect evidence* of dog occupation included the presence of dog feces, paw prints, claw marks, and remains of scavenged food. Identified caves included individual dwellings as well as series of interconnected cave structures that occurred within two-meter distance of one another. All observed dogs were photographed, and the photographs were subsequently compared and analyzed to prevent double counting.

All data were recorded using data forms that we developed for the unique purposes of this study on the World Veterinary Service (WVS) mobile phone application (23). These forms were designed to collect and georeference data pertaining to direct sightings of individual live or dead dogs, animal packs, litters, locations, and qualities of associated caves, along with multiple types of indirect evidence as described above. Notably, the WVS app can be used offline, which was valuable considering internet and mobile signals were unavailable throughout the majority of our study area.

### Cave data characterization

We estimated the density of caves stratified by occupancy status and the density of dogs stratified by age and alive status. For each visit, we calculated the mean number of dogs, stratified by age and alive status, as well as the proportion of caves showing signs of occupancy, stratified by the type of indirect evidence observed. We took pictures of observed dogs and reviewed them manually in our lab to prevent double-counting. All data handling and characterization were conducted in R (24).

### Dietary analysis

Feces were collected and geolocalized across the study region between March 1st and 4th of 2022. A dietary analysis was performed on 114 samples. Feces were washed in a bowl, and macroscopic content was left out to dry. Samples were then roughly sorted into grossly appreciable categories, including bone, fur, feather, eggshell, claw, plant fiber, unidentifiable organic matter and inorganic matter. Contents in each category were enumerated and skeletal and dental remains were identified by an individual with training in Andean zooarchaeology (K.M.). Prior to analysis, a list of common, morphologically distinct, indigenous, introduced, and domestic fauna in the Alto Selva Alegre District was compiled (Supplement 1) and served as a comparative list for osteological identifications. Based on the quality of digested bone, elements were identified based on representative taxa and skeletal elements. Remains that were highly damaged, fragmented, or otherwise non-diagnostic were labeled as unidentifiable.

## Results

### Cave and dog findings

**Figure 2:**
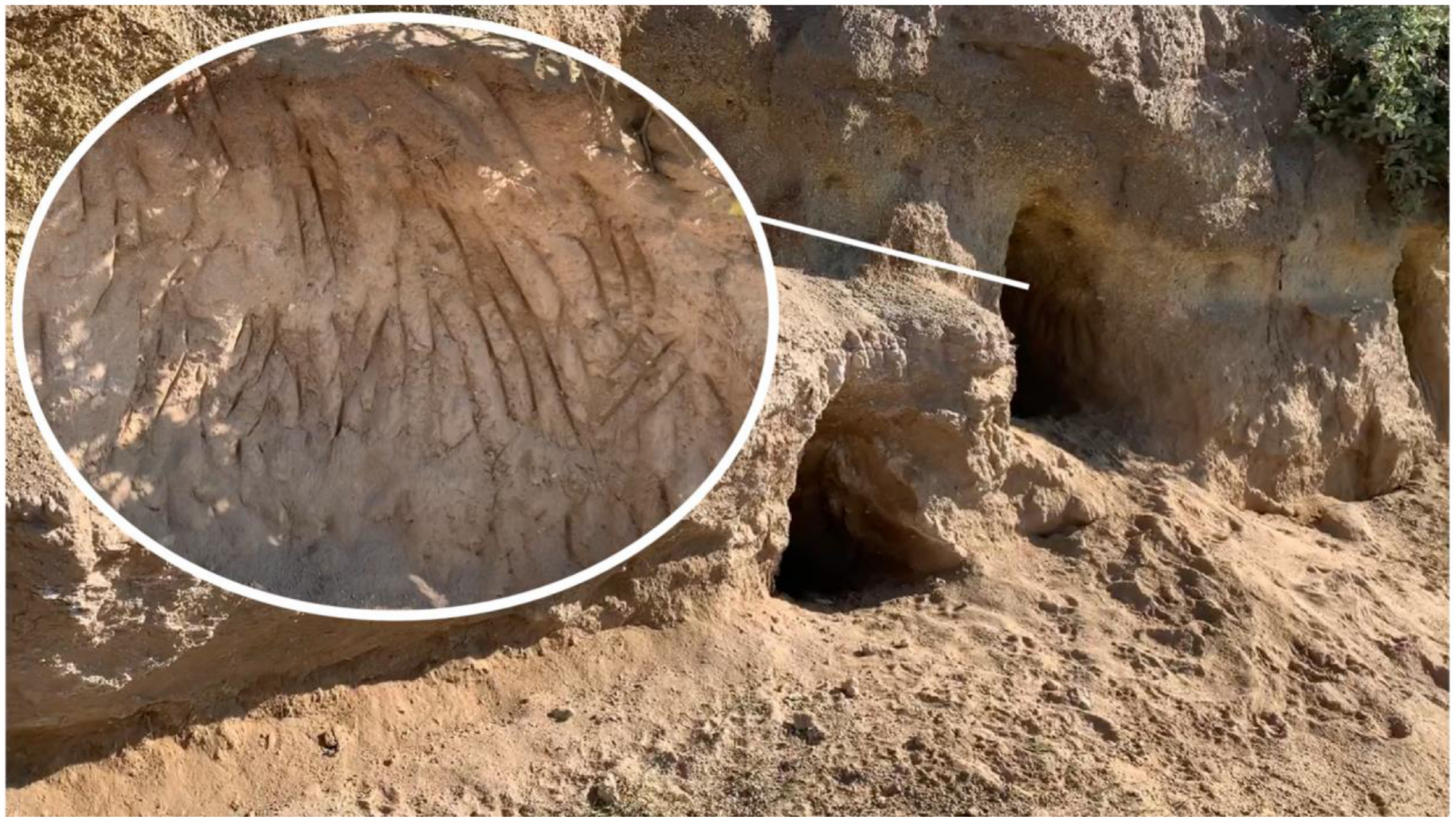
Groups of dog caves in a hill within the study area. Inset shows claw marks on the cave walls.

In 2022, we identified 38 new caves, while 61 of the original caves had collapsed and could no longer serve as dog shelters, resulting in a total of 135 caves throughout the 1.6 km^2^ study area. Many caves showed claw marks suggesting they were created or expanded by dogs (Figure 2). On average, there were 98.8 caves per square kilometer in the study area, with one locality having 133.3 caves per square kilometer (Table 1). All but 1 cave (99.4%) exhibited either direct or indirect evidence of dog use during each of the consecutive monthly visitations. Furthermore, 7.9% of these caves exhibited direct evidence of their occupation by dogs, meaning that dogs were observed either inside the cave or directly surrounding the cave at the time of the survey.

**Table 1:** Parameters of caves and dogs found in the northeastern outskirts of Arequipa city, Peru.

	**Overall**	**APSIL**	**San Luis A**	**El Roble**
Study areas	1.60 km^2^	0.73 km^2^	0.39 km^2^	0.48 km^2^
Cave density	98.75/km^2^	72.60/km^2^	133.33/km^2^	110.42/km^2^
Cave with direct evidence density	7.81/km^2^	10.27/km^2^	8.97/km^2^	3.13/km^2^
Cave with indirect evidence density	61.25/km^2^	30.14/km^2^	103.21/km^2^	74.48/km^2^
Density of live adult dogs	20/km^2^	26/km^2^	9/km^2^	20/km^2^
Density of dead dogs	9/km^2^	11/km^2^	3/km^2^	10/km^2^
Density of puppies	9/km^2^	15/km^2^	3/km^2^	3/km^2^

Over the course of four monthly visits, we observed a total of 128 live adult dogs, 55 dead adult dogs, and 17 litters comprising 55 puppies. Fieldworkers noted that the vast majority of the puppies appeared to be female, although individual-level data on sex were not systematically recorded. On average per visit, we observed 32 live adult dogs, 13.7 puppies, and 13.7 dead adult dogs, with monthly counts varying across visits (Table 1). Notably, some dead dogs were found in tight spatial clusters. Litters were detected in two of the three study localities during each round of data collection. Additionally, no dead puppies were observed during any of the four visits.

## Discussion

Our field investigations confirm the presence of feral, cave-dwelling dog populations in peri-urban areas bordering the city of Arequipa, Peru—an observation consistent with previous reports in Arequipa, and a problem that has been reported in other countries in the Americas (9, 12, 14, 25). Here, we provide new quantitative, demographic, and dietary data that substantiate and expand upon those findings. We identified feral dog populations inhabiting caves in the southeastern peri-urban outskirts of Arequipa, particularly in Alto Selva Alegre District. Following the survey presented in this study, we conducted exploratory visits to three additional districts surrounding the city and observed similar conditions—hundreds of caves inhabited by feral dogs. The presence of these dogs challenges previous assumptions that urban dog populations are largely isolated from wildlife disease reservoirs and unmanaged canine populations (7, 26).

Scat analysis, a widely used and cost-effective method for studying carnivore diets in free-ranging populations, provided robust qualitative and semi-quantitative evidence of the main taxa consumed by these dogs, even when direct observation was impractical (27). To reduce bias from differential digestibility and from the challenge of distinguishing scavenging from predation, we combined macroscopic identification with expert consultation and cross-referenced remains against local fauna lists. While scat analysis cannot capture every dietary detail, it was sufficient for our ecological and epidemiological objectives and offered unique insights into the subsistence strategies of these populations. Our dietary analysis of 194 fecal samples revealed evidence of a varied and opportunistic diet, highlighting that these cave-dogs are closely linked to human communities and other dog populations, creating additional pathways for rabies transmission beyond the vaccinated owned-dog population. Almost half of all recovered skeletal elements were unidentifiable—a proportion consistent with expected bone degradation from digestion and gnawing (28). The avian remains likely include domestic chickens, as suggested by the size and morphology of claw sheaths, skin, and bones. Informal conversations with local residents further support the notion that feral dogs hunt backyard livestock in addition to scavenging from household garbage (29–31), which is frequently discarded into dry riverbeds due to lack of formal waste management. Notably, canine skeletal remains comprised over one-third of all identifiable bones. Several fecal samples contained phalanges and metapodial elements from medium- to large-sized dogs, suggesting possible scavenging or intra-species predation—behaviors that may indicate nutritional stress or limited food availability. Although local accounts describe feral dogs hunting cats and other dogs in packs, further behavioral observation is needed to clarify whether these animals primarily scavenge (8, 32) or actively prey on conspecifics (33) and other species. Reliance on both anthropogenic resources and hunting of domestic or wild animals demonstrates that these dogs can persist independently in marginal habitats, supporting their potential role as reservoirs of infection.

Canine rabies remains endemic in the city of Arequipa despite ongoing control and elimination efforts (34, 35). Although suboptimal vaccination coverage among owned dogs is the primary factor sustaining transmission (36), unrecognized or unsurveyed subpopulations—such as feral, cave-dwelling dogs—may also contribute to the persistence of the virus (14). As the region approaches elimination thresholds, the relative importance of these overlooked groups is likely to increase. Rabies elimination strategies hinge on achieving and maintaining 70% vaccination coverage across the entire at-risk canine population (37). However, most vaccination campaigns are designed to reach owned dogs within established administrative boundaries, potentially excluding adjacent feral populations that remain unvaccinated and unsurveyed (3). Furthermore, these mass dog vaccination campaigns are only parenteral delivery, facing inherent limitations when targeting inaccessible or elusive populations such as feral cave-dwelling dogs. Our findings highlight the need to complement existing approaches with alternative tools that can extend coverage to inaccessible groups. Oral rabies vaccination (ORV) represents one promising strategy that has been successfully implemented in wildlife reservoirs and is gaining traction as a feasible option for dog populations with limited human contact (38). In the context of Arequipa, deploying ORV baits in peri-urban and cave-dwelling habitats could increase vaccination coverage among feral dogs that are unlikely to be captured or handled during conventional campaigns. Integrating ORV with community-based surveillance, humane dog population management, and ecological monitoring would strengthen a One Health approach, addressing both the epidemiological and environmental dimensions of rabies control.

Our findings underscore the spatial and ecological connectivity between urban, peri-urban, and rural dog populations, highlighting the need for expanded surveillance and control efforts that include feral dog groups. The presence of these unsurveyed populations may undermine progress toward regional and global rabies elimination goals (39). While the expansion of rabies in southern Peru has largely been attributed to human migration and the translocation of owned dogs (40), the widespread presence of feral dogs across the Arequipa region suggests that their potential role in disease persistence and spread warrants consideration. Notably, among the observed litters, the vast majority of puppies were female—an unusual deviation from the expected 1:1 sex ratio. Informal conversations with local residents indicated that people, including dog vendors, frequently visit the area to take male puppies, either for sale in local markets—where male dogs are preferred—or for use as guard animals. This ongoing removal and movement of unvaccinated puppies from unmonitored populations into the city presents an overlooked risk that should be considered in the design and implementation of rabies control programs.

In addition to their potential role in rabies epidemiology, the presence of these dogs has other important implications for public health. Settlements within a few hundred meters of these caves report some of the highest rates of dog bites globally, yet display low rates of post-exposure prophylaxis and healthcare-seeking behavior (41). While residents do not distinguish between city dogs and feral cave dogs in reports of bites, the spatial proximity and possible movement between these populations complicate surveillance and control efforts.

Worldwide, feral dogs also pose threats to local economies and biodiversity. Their presence is often first reported through farmers' and herders' complaints about livestock attacks (29–31). In ASA and surrounding districts, small-scale animal husbandry remains a key economic activity. Repeated predation on domestic animals undermines household income and food security. Moreover, wildlife authorities have documented feral dog predation on protected species, including South American camelids and Andean condors (42, 43). The potential for interspecific competition and disease transmission with native carnivores such as the Andean fox is a further concern. Feral dogs may act as reservoirs for pathogens such as canine distemper virus and sarcoptic mange, both of which can cause substantial mortality in wild canid populations (44, 45).

This study provides the first systematic documentation of feral dogs inhabiting caves in peri-urban Arequipa, highlighting an overlooked ecological niche with major implications for rabies control. A notable strength is the combination of direct and indirect evidence (photographs, sightings, feces, tracks, and food remains) to validate cave occupation, which increases confidence in the observations. The georeferencing of cave sites adds spatial epidemiological value, enabling future surveillance and interventions. Finally, the study addresses an urgent operational gap for public health programs, bridging ecological fieldwork with zoonotic disease control. The study also has limitations. First, it was observational and we did not measure rabies virus circulation limiting the ability to infer the direct role of cave dogs in transmission. Second, the sample size and geographic scope were restricted to a subset of peri-urban Arequipa, which may not capture the full variability of dog ecology across the city or other regions. Third, while photographic verification reduced double counting, individual identification methods such as tagging or genetic sampling were not used, leaving potential for misclassification. Lastly, the reliance on indirect signs of cave occupation (e.g., feces, paw prints) introduces some uncertainty, as these may persist after dogs abandon a cave.

Despite these limitations, the findings underscores the need for coordinated, cross-sectoral strategies to address the ecological and public health impacts of feral dog populations—an approach that has advanced slowly in Arequipa (46). Surveillance programs targeting cave-dwelling dogs are essential to understanding their movements, population dynamics, and role in zoonotic disease transmission. Sterilization and vaccination efforts could help reduce their ecological impact and interrupt rabies transmission chains. However, high population turnover remains a significant barrier to the long-term effectiveness of these interventions. Importantly, this challenge is not limited to feral populations: high turnover is also observed among owned dogs in Arequipa, particularly in periurban communities (47). Although we did not record any dead puppies during our surveys, this absence should not be interpreted as evidence of low pup mortality. Puppies are more difficult to detect due to their smaller size, greater use of dens, and rapid removal by scavengers or conspecifics. High juvenile mortality has been reported consistently in stray and feral dog populations, and our cross-sectional approach was not designed to track changes in litter size or survival over time. Future research using longitudinal monitoring of identified litters is needed to accurately estimate juvenile mortality, a critical parameter for modeling population turnover and rabies transmission dynamics. However, the high mortality observed among feral dogs may not represent a stable demographic trend. Reports from residents indicate that periodic poisoning or culling events occur in response to perceived threats posed by feral dog packs. Combined with the likely high rate of dog abandonment in peri-urban areas, these human-mediated factors may sustain the population despite insufficient reproduction. In areas like Arequipa city, vaccinated and sterilized feral dogs may be rapidly replaced by unvaccinated, intact dogs—often due to abandonment from nearby periurban neighborhoods. Establishing the prevalence of infectious diseases such as rabies, distemper, and mange in these feral and periurban owned dog populations will inform both wildlife conservation efforts and the design of effective public health interventions (48–50). Ultimately, controlling rabies and mitigating other health and environmental risks posed by feral dogs in peri-urban Arequipa will require adaptive, integrated One Health strategies (51). The One Health approach has been used successfully to implement cross-sectoral vaccination and surveillance programs in Africa, Asia, and the Americas to reduce dog-mediated rabies (52–54, 56), and it is a pillar of the global Zero by 30 initiative led by WHO, FAO, OIE, and partners (39, 55). By documenting the presence, ecology, and epidemiological significance of feral cave-dwelling dogs in Arequipa, our study contributes to this integrative perspective, highlighting an overlooked interface between domestic animals, wildlife, and human populations that must be addressed to achieve sustainable rabies elimination.

## Data Availability

The original contributions presented in the study are included in the article/Supplementary material, further inquiries can be directed to the corresponding authors.
