# Supplementary material for: Cave dogs around major urban areas of Arequipa, Peru, threaten rabies elimination program
Source: Front Vet Sci. 2025 Nov 19;12:1649737. doi: 10.3389/fvets.2025.1649737 (PMC12673202; doi:10.3389/fvets.2025.1649737)
Supplement: Supplementary file 1 [file Table_1.docx]

**Supplement 1. Animal Species list reported in the district of Alto Selva Alegre, Arequipa, Peru:**

Domestic animals:

- Mammals:
  - Dogs
  - Cats
  - Pigs
  - Sheep
  - Goats
- Birds:
  - Chickens
  - Rock Doves
  - House Sparrows
  - Ducks
  - Geese
- Rodents
  - Guinea pigs
  - House mouse
  - Rats
  - Rabbies

Wild Animals:

- Mammals
  - Andean fox
  - Mountain lions
  - Many species of bats
- Rodents
  - Wild mice
  - Viscacha
  - Chinchillas
- Birds
  - Birds of Prey
    - Burrowing owl
    - Black vulture
    - Turkey vulture
    - American Kestrel
    - Multiple hawks
    - Mountain caracara
    - Black-chested buzzard eagle
  - Passerine Birds
    - Sparrows
    - Finches
    - Thrushes
    - Conebills
    - Siskins
  - Hummingbirds
  - Wading birds
    - Black-crowned night heron
    - Cattle egret
    - ibises
  - Cormorants
  - Chilean flamingo
  - Doves
  - Ground birds
    - Peruvian thick-knee
- Reptiles
  - Peruvian Pacific iguana
  - Peruvian slender snake
  - Tschudi’s Pacific iguana
  - Haseman’s gecko
- Amphibian
  - Chili water frog
  - Warty toad
