# Supplementary material for: Cave dogs around major urban areas of Arequipa, Peru, threaten rabies elimination program
Source: Front Vet Sci. 2025 Nov 19;12:1649737. doi: 10.3389/fvets.2025.1649737 (PMC12673202; doi:10.3389/fvets.2025.1649737)
Supplement: Supplementary file 2 [file Table_2.docx]

**Supplement 2**

**Dietary Analysis**

We analyzed 194 fecal samples collected from caves. Recovered materials included beaks, bones, claws, feathers, plant fibers, keratin, plastic fragments, eggshells, skin, and teeth (S1). The majority contained skeletal remains, allowing taxonomic identification in 57 samples. The most frequent identifiable items were birds (44%), dogs (37%), and sheep (9%), followed by rodents (7%) and cats (3%). Several bird and rodent remains were consistent with domestic species such as chickens and guinea pigs, which are common in backyard livestock systems. Other specimens were suggestive of additional wild species, but conclusive identification was not possible.

**Table 2.** Dietary analysis from feces collected in and around caves inhabited by feral dogs in Arequipa, Peru, in 2022.

| **Material** | **Count (n=194)** | **Percent** | **95% CI** |
| --- | --- | --- | --- |
| Bone | 100 | 51.55% | 44.28 - 58.76 |
| Claw | 27 | 13.92% | 9.38 - 19.60 |
| Fiber | 26 | 13.40% | 8.95 - 19.02 |
| Feather | 18 | 9.28% | 5.59 - 14.27 |
| Teeth | 8 | 4.12% | 1.80 - 7.96 |
| Eggshell | 7 | 3.61% | 1.46 - 7.29 |
| Skin | 5 | 2.58% | 0.84 - 5.91 |
| Beak | 1 | 0.52% | 0.01 - 2.84 |
| Keratin | 1 | 0.52% | 0.01 - 2.84 |
| Plastic | 1 | 0.52% | 0.01 - 2.84 |
